# Operationalizing a model to quantify implementation of a multi-component intervention in a stepped-wedge trial

**DOI:** 10.1186/s13012-018-0720-2

**Published:** 2018-02-08

**Authors:** Linnea Ferm, Charlotte Diana Nørregaard Rasmussen, Marie Birk Jørgensen

**Affiliations:** National Research Centre for the Working Environment, Lersø Parkallé 105, DK-2100 Copenhagen, Denmark

**Keywords:** Stepped-wedge design, Process evaluation, Pragmatic trials, Fidelity, Receipt, Delivery

## Abstract

**Background:**

It is challenging to interpret the results of multifaceted interventions due to complex program theories that are difficult to measure in a quantifiable manner. The aims of this paper were, first, to develop a model for a comprehensive quantitative implementation evaluation and, second, to operationalize it in the process evaluation of the stepped-wedge cluster randomized controlled trial: “Prevention of low back pain and its consequences among nurses’ aides in elderly care” to investigate if implementation differed across intervention components, steps, and settings (workplaces).

**Methods:**

Operationalization of a quantifiable measure of implementation requires three steps: (1) development of a program logic and intervention protocol, (2) description of a complete and acceptable delivery of the intervention, and (3) description of what determines the receipt of the intervention. Program logic from a previously developed multifaceted stepped-wedge intervention was used. The optimal delivery of the intervention was defined as the deliverers’ full understanding and following of the intervention protocol and that they performed their best and contributed to the participants’ attention and motivation (fidelity). The optimal receipt of the intervention was defined as participants being fully present at all intervention activities (participation), being motivated and satisfied, and having a good social support (responsiveness). Measurements of the fidelity, participation, and responsiveness were obtained from logbooks and questionnaires. Fidelity was multiplied by participation to measure exposure of the intervention to the individual. The implementation was determined from optimal delivery and optimal receipt on a scale from 0 (no implementation) to 100 (full implementation) on individual and organizational level.

**Results:**

Out of 753 sessions, 95% were delivered. The sessions were delivered with 91% success (fidelity) across the organization. Average participation, fidelity, exposure, and responsiveness were 50, 93, 48, and 89% across all participants. The implementation of the intervention was uniform across steps (*p* = 0.252) and workplaces (*p* = 0.125) but not for intervention components (*p* = 0.000). However, participation, fidelity, exposure, and responsiveness varied between workplaces.

**Conclusions:**

This study developed a quantifiable implementation evaluation measuring participation, fidelity, exposure, and responsiveness. The quantifiable implementation evaluation was suitable for comparing implementation across steps, components, and settings and can be applied in the analyses on the impact of implementation of complex interventions.

## Background

More and more public health interventions consist of multiple intervention components, are performed in complex settings and social structures with often heterogeneous populations and build on complex program theories. These characteristics challenge the interpretation of classic rigid evaluations in randomized controlled trial designs since a mere effect evaluation only explains a fraction of the causal assumptions in the program theory [1, 2]. Furthermore, the effect evaluation itself risks rejecting the hypothesis underlying the program theory, due to implementation failure (type III error) [1].

Therefore, process evaluations have been increasingly developed, debated, and used. Previously, process evaluations were mostly anecdotal and were mainly used as formative analyses rather than summative [3]. However, in recent years, a number of useful frameworks have been developed to guide researchers’ evaluation plans [2, 4–7]. For example, Sanchez et al. [8] offered a comprehensive guide to develop a process evaluation plan that covered both formative and summative applications of different process measures (fidelity (implemented as planned), dose, reach, recruitment, and context) [8]. Furthermore, they illustrate its use in a proposed school-based case study. However, only the process evaluation plan is presented––not the operationalized process evaluation in actual values or results [8]. Linnan and Steckler [5] proposed similar process measures and had concrete suggestions as to how to quantify implementation. Nevertheless, still only few process evaluations offer the opportunity to compare process outcomes between studies or to use process outcomes in mediation and/or moderation analyses to understand which of the intervention components affect the intervention outcome [6]. For the latter, particularly level of implementation of the intervention is useful to evaluate in a quantifiable manner. Multiple determinants like the program characteristics (size, coverage, and complexity), setting characteristics, facilitation strategies, reach of participants (fraction of all eligible), and the participants’ motivation are potential predictors of the implementation and are therefore also important factors to measure and include in process evaluations [2, 6, 9, 10]. However, most central to the studies’ effectiveness evaluation is whether or not the intervention was implemented as planned; and therefore, the quantifiable assessment of implementation deserves specific attention.

Implementation has been suggested to be the sum of the dose and quality of delivery of the intervention (fidelity) and the receipt of the intervention in a number of frameworks and checklists [3–6, 11]. The receipt is often measured merely as participation; however, responsiveness is also a factor in receipt of the intervention [12]. It explicitly measures factors that impede or hamper the manifestation of the intervention effect within the individual and may therefore be important to consider as a mediator, highly proximal to the intervention [12]. Implementation is however complex to assess when the intervention consists of multiple key effective components [6, 13]. Furthermore, balancing fidelity against the opportunity for adaptability of the intervention content to the receivers can be challenging [1]. Furthermore, no one has to our knowledge provided a meaningful step by step operationalized guide of how to quantify implementation in multicomponent interventions. Therefore, we developed a model with a step-by-step operationalized guide to conduct a comprehensive quantitative implementation evaluation and operationalized it in a multifaceted stepped-wedge intervention delivered to four workplaces in Denmark. The model and the step-by-step operationalized guide are highly inspired by the methods suggested by Linnan and Steckler [5], Caroll et al. [6], and Saunders et al. [4] but offer specific values for implementation that will be comparable between studies and useful to detect the most effective components in the intervention. Also, the model addresses the challenge of how to quantify the variable participation of individuals over learning sessions across sites and clusters in which the intervention is implemented. Furthermore, the model offers opportunity to deal with the implementation issues particularly evident in stepped-wedge designs, where the intervention is implemented to randomized groups in a phased manner, and there is a risk that implementation changes over time (i.e., with increased experience among intervention deliverers) which challenges the interpretation of the effect analyses [14]. Thus, the quantification of implementation may be vital in building an evidence base that informs policy and practice [2]. Therefore, the aim of this paper was, first, to develop a model for a comprehensive quantitative implementation evaluation and, second, to give a step-by-step guide by operationalizing it in a multifaceted stepped-wedge intervention implemented in four workplaces and to investigate if implementation differed across intervention components, delivery timings (trial steps), and the workplaces (settings) involved.

## Methods

In the development of a model for a comprehensive quantitative implementation evaluation, we have carefully reviewed existing theories and process evaluation frameworks and models [4–6]. Hence, the current model is not a new theory in itself or a new view on implementation, but a quantitative operationalization of previous theories.

### Development of the model of how to quantify implementation in multicomponent interventions

Previous theories and frameworks [4, 5] suggest that implementation consist of two overall aspects: (1) *delivery of the intervention* (dose, content (the amount and proportion), and quality of the intervention that are delivered as planned) and (2) *receipt of the intervention* (the amount received and perceived by the participants as intended). To be able to develop a quantifiable implementation measure, it is important to be particularly specific regarding these aspects. Thus, the following three steps aim to guide which aspects need to be particularly carefully considered for the implementation model to work: (1) development of a program logic and an intervention protocol, (2) define what determines an optimal delivery of the intervention, and (3) define what is an optimal receipt of the intervention.

1. Development of program logic and intervention protocol

First, a program logic must be developed describing the purpose of the program, the objectives, the change process, the expected impact, and outcome of the intervention. The program logic should be supported by an operationalized program logic––a written intervention protocol for the intervention deliverers, that specifies:

- Which are the effective components?
- How important is fidelity compared with adaptation? That is, should the protocol be strictly followed or is there room for adapting the content to the target group, and if yes, how much?

2) Description of complete and acceptable delivery of the intervention

Elements of the intervention that need to be delivered during the study to preserve treatment integrity should be defined as pre-specified success criteria for each of the intervention components for each of the sessions and written in the intervention protocol. With the clear definition of the success criteria, also opportunities for adaptability of the intervention content to the receivers must be clarified. Finally, the success criteria should be measurable (i.e., so that questions for measuring the pre-specified success criteria can be developed).

3) Description of what determines receipt of the intervention

Particularly for the receipt of the intervention, it is highly up to the developers of the program logic to define what are the key components that determine when an individual has received an intervention. For example, an intervention may happen at a higher organizational level, i.e., at team level. Then, what determines how much the individual is exposed to the intervention? A very simple measurement of receipt of the intervention, often used in previous implementation evaluations, is participation or attendance [15]. However, it must be pre-defined by the researchers which degree of participation is needed and whether for example co-worker/peer participation contaminate others? However, apart from the mere presence of a participant, also responsiveness to an intervention, may determine how much of the intervention is taken up by the individual. Factors previously suggested to impact responsiveness may be motivation for the intervention, satisfaction, engagement, pre-intervention expectations, knowledge, and social support during the intervention [12]. In some instances, such factors may have a very small and perhaps negligible role (for example, in case of a merely structural intervention) and in some cases, it may have a highly determining role (for example, in case of a choice-based intervention). The role of the responsiveness must be considered and made measurable before the implementation can be quantified.

### The complex multifaceted stepped-wedge intervention

For the current example study, the program logic has been reported in a research protocol [16]. We designed a multifaceted intervention for the prevention of low back pain (LBP) among nurses’ aides in elderly care in Denmark evaluated in a stepped-wedge randomized controlled trial. The intervention was built on a comprehensive theoretical framework of both effectiveness and implementation in the development and planning of the intervention [17] and used a participatory approach that involved all levels of the organization (management, supervisor, workers). In short, the intervention lasted 3 months and consisted of a combination of participatory ergonomics (PE), physical training (PT), and cognitive behavioral training (CBT) and was conducted in the teams at the workplace. PE consisted of a kick-off meeting for all participants where an ergonomic working group was formed that participated in two workshops and two evaluation meetings. The PE was conducted as an organizational intervention meaning that only a subgroup of the participants was to participate in the workshops and evaluation meetings and that contamination to the rest of the team was expected. CBT consisted of two workshops of 3-h duration for all participants. PT consisted of 12 weekly 1-h sessions for all participants. In total, the intervention consisted of 19 sessions/27 h (PT (12 sessions/12 h), CBT (2 sessions/6 h), and PE (5 sessions/9 h)). The intervention was conducted during paid working hours and delivered by six local trained physiotherapists and occupational therapists (deliverers). The optimal delivery of the intervention required that the deliverers fully understood and followed the intervention protocol that they performed their best and that they contributed to the workers’ attention and motivation. An optimal receipt of the intervention required that the participants were present at all PT sessions, and all CBT sessions and that their work team was present at all PE sessions and that they were motivated, satisfied with the interventions, and had a perception of high support during the sessions. The multifaceted intervention was conducted in 2013/2014 and is described in detail in Rasmussen et al. 2013 [16], and the effect on LBP is reported in Rasmussen et al. 2015 [18].

The workplaces, which were recruited to participate in the trial (adoption), and the employees, who were chosen to be enrolled in the trial (reach) have been described previously. In short, 9 workplaces (districts) in a municipality were offered participation. Four of the nine workplaces in the elderly care administration of the municipality adopted the project corresponding to 44%. There were 1074 eligible employees from 58 teams and of those 594 wanted to participate and were randomized to the intervention [19]. The participants were randomized to 4 groups beginning the intervention at 4 different time points, with 126 participants in group 1, 146 participants in group 2, 158 participants in group 3, and 164 participants in group 4. The intervention components were to be delivered equally to all teams in the four workplaces.

### Measures

The data were gathered from questionnaires for the participants and from deliverer logbooks (Table 1). Moreover, in order to check the quality of the deliverers’ responses to the logbooks, observations of random sessions were conducted. The observations were all conducted by one observer, and the observation guide included the exact same questions as the questions posed in the deliverer logbooks.

**Table 1:** Overview of data sources, questions, and response categories that constitute each of the concepts that are involved in the implementation (delivery (content and quality) and receipt (participation and responsiveness)). All response categories were scored into a scale from 0 to 100 (numbers given in parentheses after each of the response categories)

	Data source	Question	Response categories
Delivery (content and quality)			
Content (success criteria)	Instructor logbooks	Have you implemented the following according to the manual: (55 success criteria)?	Not implemented (0)/partly implemented (50)/completely implemented (100)/implemented more in depth (100)
Quality (understanding of the day’s theme)	Instructor logbooks		
		To which extent...	To a very large extent (100)/to a large extent (100)/some-what (50)/to a small extent (0)/to
		Has the activity of the day (as described in the protocol) been very small extent (0) easy to comprehend? ...did you have a clear understanding of today’s theme(s)?	
		…do you think today’s themes were relevant?	
		...do you think today’s themes was interesting?	
Qaulity (contribution to the participants’ learning)	Instructor logbooks		
		Regarding today, to which extent have you contributed to…	To a very large extent (100)/to a large extent (100)/somewhat (50)/to a small extent (0)/to a very small extent (0)
		...the participants’ commitment and motivation? ...ensuring the employees’ participation in the activity? ...adapting the activity to the needs of the participants?	
		...maintaining the participants’ attention?	
Quality (self-rated performance)	Instructor logbooks		
		Suppose that your performance, at its best, is equal to 10 points. How would you rate your performance today?	0–10 (0 = not capable to perform; 10 = best performance) (0–4 were scored 0, 5–7 were scored 50, 8–10 were scored 100)
Receipt (participation and responsiveness)			
Participation	Instructor logbooks		
		Attendance at session	Yes/no (0–100)
Responsiveness	Intervention evaluation questionnaire, completed by the participants		
		To which extent have you...	To a very large extent (100)/To a large extent (100)/Some-what (50)/To a small extent (0)/To a very small extent (0)
Responsiveness (Satisfaction)		...been satisfied with the physical training? ...been satisfied with the cognitive training? ...been satisfied with the ergonomics? ...been satisfied with project overall? ...all in all, found the project relevant? ...all in all, found the project interesting?	
Responsiveness (intervention-related social support)		The instructor supported and encouraged me?	Often (100)/always (100)/sometimes (50)/seldom (0)/never (0)
		The group supported and encouraged me?	
		I trusted the instructor and shared my own challenges and thoughts?	
		I trusted the group and shared my own challenges and thoughts?	
Responsiveness (motivation)	Instructor logbooks	To which extent are the participants committed and motivated?	To a very large extent (100)/to a large extent (100)/somewhat (50)/to a small extent (0)/to a very small extent (0)

#### Delivery of the intervention (fidelity)

##### Dose

The number of sessions delivered compared to the number of sessions that were supposed to be delivered during the entire intervention was registered.

##### Content (success criteria)

To measure if the deliverers followed the intervention protocol, the success criteria within each component for each session were measured by asking the deliverers to fill in a logbook after each session. The logbooks were specifically developed for each type of session to match the success criteria for that specific session. It was explained to the deliverers that the logbooks were for analyses purposes only and possible poor ratings of one’s own performance as a deliverer would not be criticized or used against them. All in all, 55 questions were included in the logbooks over the course of all sessions. The questions were “Have you implemented the following according to the manual: (success criteria)?” with (success criteria) substituted with the specific success criteria for each part of the session (i.e., “generated ergonomics working groups?”). The response categories for all the success criteria questions were “not implemented,” “partly implemented,” “completely implemented”, and “implemented more in depth.” The last response category was included to contribute to possible more in depth understanding of the implementation since more in depth implementation of one theme may compensate for less implementation of another theme [8]. However, the “implemented more in depth” option is not used in the calculation of the implementation. Therefore, answers “completely implemented” and “implemented more in depth” on the 4-point scale were scored 100, “partly implemented” was scored 50, and “not implemented” was scored 0. Specifically, for the PT sessions, the extent the PT fulfilled the success criteria also considered the physical intensity of the sessions. The intensity was evaluated by the instructor in the instructor logbooks measured as the maximal intensity during the PT at a group level on a scale ranging from 0 to 10. The scale was scored as 0–3 = 0, 4–5 = 50, 6–7 = 75, and 8–10 = 100.

##### Quality (deliverers’ performance)

The quality of the intervention was measured as the deliverers’ performance in terms of understanding, contribution, and overall self-rated performance in the logbooks [8]. Understanding and contribution to the participants’ learning both consisted of four questions on a 5-point scale with response categories: To a very large extent/to a large extent/somewhat/to a small extent/to a very small extent. The four questions posed concerning understanding were “To which extent (i) has the activity of the day (as described in the manual) been easy to comprehend? (ii) Did you have a clear understanding of today’s theme(s)? (iii) Do you think today’s themes were relevant? (iiii) Do you think today’s themes were interesting?”. The four questions concerning contribution to the participants’ learning were: “Regarding today, to which extent have you contributed to (i) the participants’ commitment and motivation? (ii) Ensuring the participants’ participation in the course? (iii) Adapting the course to the needs of the participants? And (iiii) maintaining the participants’ attention?” The deliverers’ self-rated performance was measured by one question: “Suppose that your performance, at its best, equals 10 points. How would you rate your performance today?” The response categories were on a scale from 0 to 10, where 0 corresponds to “not capable to perform” and 10 corresponds to “best performance.” For the 5-point scale, the two most positive answers were scored 100, the two most negative answers were scored 0, and the answer in the middle was scored 50. The 0–10 scale regarding the deliverers’ self-rated performance was scored as follows: 0–4 = 0, 5–7 = 50, and 8–10 = 100.

##### Exposure

The uptake of the intervention is dependent on whether the participant attends a session or not and on the fidelity of the sessions that the participant attended. Therefore, exposure was calculated as the sum of the fidelity of attended sessions (see more in calculation).

#### Receipt of the intervention

##### Participation

For all sessions in PT and CBT, participation was calculated based on individual presence at a session (yes/no) weighted according to the duration of the session registered through the deliverers’ logbooks. This was also done for the kick-off meeting, where all were invited to participate. However, in the four sessions of PE, where only a subgroup was participating, the participation was calculated on team level based on supervisor affiliation, and the participation for each team were assigned to each worker of the specific team. This was to allow that all workers were given participation since the PE was anticipated to affect all workers within a team (contamination). The participation for the PE was calculated as a combined participation score and continuity score. The participation score was the percentage of participants, according to a required number of representatives weighted according to the duration of the session. A supervisor with less than five workers were required to have two representatives participating in the sessions, a supervisor with 5–9 workers was required to have three representatives in the sessions, a supervisor with 10–14 workers was required to have four representatives in the sessions, a supervisor with 15–19 workers was required to have five representatives in the sessions, and a supervisor with 20 or more workers was required to have 6 representatives in the sessions. The continuity score was calculated as the team participation score with an exception that the score was dependent of whether it was the same representatives participating in all the sessions since it was anticipated that continuation would improve implementation. The total percentage for the PE were calculated by summing the team participation score and the continuity score and divide it by 2, and the value was assigned to each worker of the respective team. Finally, the overall participation of all the sessions was calculated by summing the PE participation with the PT and CBT participation weighted according to the overall duration of the sessions (27 h).

##### Responsiveness

Participants’ responsiveness was measured by satisfaction, social support, and motivation [8, 12]. The participants were asked to fill in a questionnaire at the end of the entire intervention.

The questions regarding satisfaction of the intervention included six questions with responses on a 5-point scale with response categories being: to a very large extent/to a large extent/some-what/to a small extent/to a very small extent. The first three questions referred exclusively to one of the three intervention components: “To which extent have you been satisfied with (the PT/the cognitive behavioral training/the ergonomics)?” The final three questions referred to the overall intervention: “To which extent have you been satisfied with the project overall?” and “To which extent have you all in all found the project (relevant/interesting)?”

Intervention-related social support was measured in the questionnaire for the participants and included four questions on a 5-point scale with response categories being: always/often/sometimes/seldom/never. The questions posed were: (i) “The deliverer supported and encouraged me?”, (ii) “The group supported and encouraged me?”, (iii) “I trusted the deliverer and shared my own challenges and thoughts?”, and (iiii) “I trusted the group and shared my own challenges and thoughts?”

Participants’ motivation was measured by a question in the deliverers’ logbook: “To which extent are the participants committed and motivated?” The answer categories were on a 5-point scale: to a very large extent/to a large extent/some-what/to a small extent/to a very small extent.

The 5-point scale was scored with the two most positive answers as 100, the two most negative answers were scored 0, and the question in the middle was scored 50.

### Calculation of the implementation at organizational level and individual level

The calculation of implementation is based on several data extracted from the data collection. The implementation is calculated at the organizational level to describe the overall implementation of the intervention according to the protocol for the organization as a whole. Organization level implementation can be used to give an estimate of how well the organization implements the intervention. The individual level implementation gives a number to each individual participant indicating their specific level of implementation according to the protocol and can be used to analyze the association to individual outcomes. Individual level implementation is highly dependent on the organizational level implementation since, for example, the individual participant cannot gain a session, if it is canceled by the organization. However, the organizational level implementation is also dependent on the individual participants’ implementation. Therefore, the mean implementation of all individuals will be close to similar to the organizational level implementation.

#### Implementation at organizational level

For the calculation of the implementation at organizational level, three numbers are needed. First, the overall dose delivered (*D*) is calculated as the percentage of sessions delivered (*D*^*d*^) per number of sessions intended to deliver according to the protocol (*D*^*i*^):

$$ D={D}^d/{D}^i $$

For example, if the protocol is to deliver 800 sessions, but only 700 sessions are delivered, the dose would be:

$$ D=700/800=0.875\sim 88\% $$

Second, the proportion of fidelity (F) (content (C) and quality (Q)) delivered compared to the protocol is calculated. Since C and Q are measured as a percentage delivery according to the protocol, equaling 100%, the fidelity is calculated as:

$$ F=C+Q/2 $$

In an example where 50% of the content is delivered with 80% of the quality, the fidelity is:

$$ F=0.5+0.8/2=0.65\sim 65\% $$

The overall organizational level implementation would then be calculated as the average of dose and fidelity:

$$ D+F/2=0.875+0.65/2=0.7625\sim 76\% $$

#### Implementation at the individual level

Implementation at the individual level consists of fidelity, participation, and responsiveness. Since the fidelity varies from session to session, and participation is session-dependent, implementation at individual level has to be calculated at session level first.

Fidelity at session level (*F*^*s*^) is calculated as session level delivery of content (*C*^*s*^) and quality (*Q*^*s*^), where both *C*^*s*^ and *Q*^*s*^ are measured as a percentage of what was specified in the protocol, equaling 100%:

$$ {F}^s={C}^s+{Q}^s/2 $$

An individual is only exposed to the *F*^*s*^ if he or she participates at the session (*P*^*s*^). Participation at a session is either yes (=1) or no (=0) and for ergonomic sessions which are team-dependent any number between 0 and 1. Furthermore, session participation needs to be weighed according to the participation in all sessions as specified in the protocol. That is, if one participant is present at one session of 3-h duration, out of 27 h of intervention sessions in total, the participation will be *P*^*s*^ = 3/27 = 0.11.

To calculate how much fidelity the individual then has been exposed to, we calculate session level exposure (*E*^*s*^) as:

$$ {E}^s={F}^s\times {P}^s $$

That is, for example, if *F*^*s*^ = 0.65 and *P*^*s*^ = 0.11, then

$$ {E}^s=0.65\times 0.11=0.072\sim 7\% $$

This means that the individual is exposed to 7% of the total intervention during this session of 3-h duration.

However, even though the exposure is 7%, the individual responsiveness (*R*) will determine the uptake of the exposure, and therefore, the individual session level implementation (*I*^*s*^) will be:

$$ {I}^s={E}^s\times R $$

An individual responsiveness may be 80% (*R* = 0.8), and *I*^*s*^ will then be:

$$ {I}^s=0.072\times 0.8=0.0576\sim 6\% $$

If either of the factors that constitute implementation (*F*^*s*^, *E*^*s*^, *P*^*s*^, or *R*) equals 0, implementation will be zero.

Finally, to calculate the total exposure and implementation at individual level, all the exposure and all the implementation values are summed for all of the sessions, respectively.

For exposure:

$$ {E}^{\mathrm{all}}=\mathrm{Sum}\left({E}^{s1}+{E}^{s2}+{E}^{s3\dots \dots \dots .}\right) $$

For implementation:

$$ {I}^{\mathrm{all}}=\mathrm{Sum}\left({I}^{s1}+{I}^{s2}+{I}^{s3\dots \dots \dots .}\right) $$

At the end, each individual will have a session-level exposure and a session-level implementation and a total exposure and total implementation that may range from 0 to 100. Finally, a mean exposure and mean implementation can be calculated for the population as a whole and can be stratified for the different intervention components (PE, PT, and CBT), the workplaces and the delivery timing (steps of the stepped-wedge design).

**Fig. 1:**
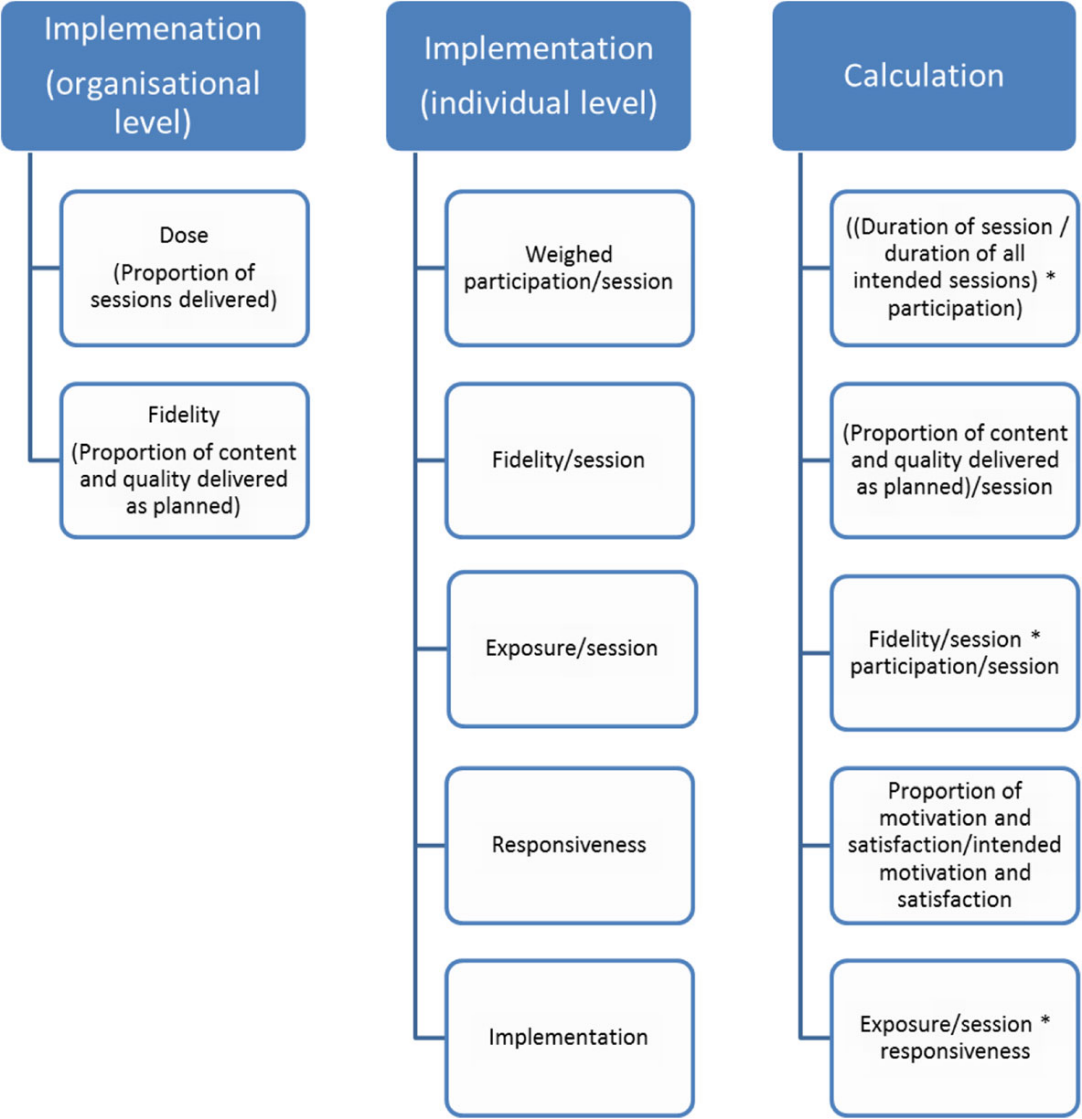
Implementation. Illustration of factors that constitute the implementation at organizational and individual level and calculation of the implementation score

The calculations are summarized in Fig. 1.

### Analyses

With Principal Component Analysis (PCA), we investigated whether we could make separate indexes for the measures regarding the quality of the intervention (understanding, contribution, overall self-rated performance) and responsiveness (satisfaction, support, motivation).

The participants who responded to the intervention evaluation questionnaire were compared to the non-responding participants with a Pearson’s chi**-**square test for categorical variables and Student’s *t* test for continuous variables.

The inter-rater reliability of the deliverer logbooks was tested against random samples of session observations by an intraclass correlation analysis supported by a Bland-Altman plot.

To investigate the degree of implementation across different factors (delivery timing (the stepped-wedge design) and workplaces), ANOVA tests were used. For post hoc analyses to determine where the groups may differ, we used Tukey or Games Howell depending on whether the data met the homogeneity of variance assumption. To investigate the degree of implementation between intervention components, we used a repeated measures ANOVA. For post hoc analyses to determine where the groups may differ in intervention components, we used the Bonferroni test.

The group responding to the intervention evaluation questionnaire (*N* = 299 (50%)) is constituted purely by those participants who were present at the last session; and therefore, it is a selected group with a much higher participation (68%) than the entire population (50%). Therefore, the analyses on the differences within the delivery timings, intervention components, and workplaces are conducted both on fidelity, participation, responsiveness, and the implementation score (only on the selected population of responders) and on the exposure score (both on the selected and on the entire population).

## Results

The PCA for quality of the intervention showed that two separate indexes could be made based on the original questions posed. The items regarding understanding constituted one index with a Cronbach alpha of 0.812, and the items regarding contribution constituted the second index with a Cronbach alpha of 0.836. Overall self-rated performance did not fit into any of the indexes and therefore constituted a measure in itself.

The PCA for the responsiveness of the intervention showed that two separate indexes could be made based on the original questions posed. The items regarding satisfaction constituted one index (Cronbach alpha = 0.862), and the items regarding social support constituted the second index (Cronbach alpha = 0.660).

There were 299 participants that answered the intervention evaluation questionnaires (responders) and 295 who did not (non-responders). Responders were significantly older (48 years) than non-responders (46 years) (*p* = 0.012), and responders had significant higher participation (68%) than non-responders (32%). No differences were found in gender, ethnicity, or LBP days at baseline. The inter-rater reliability of the deliverer logbooks tested against 44 random samples of session observations with an intraclass correlation analysis supported by a Bland-Altman plot showed a tendency towards a general overestimation of performance from the deliverers compared to the observer (data not shown).

### Delivery of the intervention

In Table 2, the dose delivered of each of the three intervention components and the total intervention at organizational level are shown. A total of 713 sessions were delivered (95%). The mean score for delivery was 99, 93, and 98% for PE, PT, and CBT, respectively. The fidelity of the sessions specified in the protocol varied from 85 to 89%, and the fidelity of the sessions held varied from 90 to 94%.

**Table 2:** Dose delivered of each of the three intervention components and the total intervention at session level according to the 753 sessions that were intended to be held and the 713 sessions that were actually held, and fidelity of intended sessions according to the protocol (fidelity × percentage of sessions held) and the fidelity of those sessions that were held

	Participatory ergonomics (*n* = 172)		Physical training (*n* = 501)		Cognitive behavioral training (*n* = 80)		Total intervention (*n* = 753)	
	*n* (%)		*n* (%)		*n* (%)		*n* (%)	
Dose delivered (sessions held/intended)	171 (99%)		464 (93%)		78 (98%)		713 (95%)	
	Mean (%)	SD	Mean (%)	SD	Mean (%)	SD	Mean (%)	SD
Fidelity of intended sessions (*n* = 753)	89	9	85	12	89	8	86	11
Content (success criteria)	94	12	80	17	94	8	84	16
Quality (performance)	87	11	88	10	87	12	88	11
*Understanding*	92	9	93	7	88	13	92	9
*Contribution*	91	12	92	11	93	6	92	11
*Overall self-rated performance*	79	22	80	23	80	22	80	23
Fidelity of sessions held (*n* = 713)	94	10	90	13	94	8	91	12
Content (success criteria)	95	12	86	18	96	8	89	17
Quality (performance)	92	12	93	11	92	12	93	11
*Understanding*	97	10	98	7	92	14	97	9
*Contribution*	96	13	97	12	98	6	97	12
*Overall self-rated performance*	84	23	85	25	84	23	84	24

The delivery and each of the components that constitute delivery are measured on a scale between 0 and 100, with 100 being the best

SD standard deviation

In Table 3, the delivery (fidelity and exposure) of each of the three intervention components and the total intervention at individual level are shown. The mean fidelity for the total intervention was 93%. The mean score for fidelity was 92, 91, and 95% for PE, PT, and CBT, respectively. Furthermore, Table 3 shows the mean exposure of individuals in each of the three intervention components and in total. The exposure for the total intervention was 48%, and 63, 34, and 45% for PE, PT, and CBT, respectively.

**Table 3:** Delivery (fidelity and exposure) and receipt (responsiveness and participation rates) for each of the three intervention components and the total intervention at individual level (weighted according to duration of sessions)

	Participatory ergonomics			Physical training			Cognitive behavioral training			Total intervention		
	*N*	Mean (%)	SD	*n*	Mean (%)	SD	*N*	Mean (%)	SD	*n*	Mean (%)	SD
Delivery												
Fidelity	583	92	10	427	91	6	353	95	7	351	93	4
Content (success criteria)	583	97	4	427	88	7	353	97	6	351	94	4
Quality (performance)	583	91	14	427	94	6	353	92	10	351	93	6
*Understanding*	583	94	13	427	98	4	353	94	12	351	96	6
*Contribution*	583	94	13	427	98	5	353	98	5	351	97	5
*Overall self-rated performance*	583	83	18	427	85	15	353	86	19	351	85	11
Exposure	594	63	22	594	34	29	594	45	42	594	48	26
Content (success criteria)	594	64	22	594	33	27	594	46	42	594	48	26
Quality (performance)	594	61	23	594	36	30	594	44	41	594	47	26
*Understanding*	594	64	23	594	37	31	594	45	42	594	49	27
*Contribution*	594	64	23	594	37	31	594	47	43	594	49	27
*Overall self-rated performance*	594	57	23	594	33	28	594	41	39	594	43	25
Receipt												
Responsiveness	224	90	10	251	91	7	224	92	9	221	89	8
*Satisfaction*	227	83	20	257	88	18	253	85	19	225	83	19
*Social support*	269	93*	14*	269	93*	14*	269	93*	14*	269	93*	14*
*Motivation*	594	91	18	427	96	6	353	93	15	594	92	16
Participation	594	66	22	594	38	31	594	48	44	594	50	26
Implementation	224	60	23	251	30	29	224	35	39	221	56	20

The delivery and receipt and each of the components that constitute these are measured on a scale between 0 and 100, with 100 being the optimal delivery and is therefore expressed in percentage

*The value is identic to the value of the total intervention, though there are no specific values for each intervention component

SD standard deviation

Table 3 also shows the receipt (participation and responsiveness) of the intervention. Responsiveness was based on measurements of those answering the follow-up questionnaire (*n* = 299). The mean responsiveness for the total intervention was 89%. The mean score for responsiveness was 90, 91, and 92% for PE, PT, and CBT, respectively. The average participation for the total intervention was 50%. The average participation for PE, PT, and CBT was 66, 38, and 48%, respectively.

The final implementation score (responsiveness (*R*) × exposure (*E*)) for the total intervention was 56%, and for PE, PT, and CBT the implementation was 60, 30, and 35%, respectively.

Table 4 shows the results of the implementation components across delivering timings, workplaces, and intervention components. For delivering timings, there were significant differences in fidelity (*p* = 0.000). For workplaces, there were significant differences in fidelity (*p* = 0.000), exposure (*p* = 0.004), participation (*p* = 0.001), and responsiveness (*p* = 0.001). For intervention components, all implementation components were statistically significant different (*p* = 0.000).

**Table 4:** Means of implementation components at individual level across delivering timings, workplaces, and intervention components

	Delivery								Receipt								Implementation			
	Fidelity			*P* value	Exposure			*P* value	Participation rate			*P* value	Responsiveness			*P* value	Implementation			*P* value
	*n*	Mean	SD		*n*	Mean	SD		*N*	Mean	SD		*n*	Mean	SD		*n*	Mean	SD	
***Total***	***351***	***93***	***4***		***593***	***48***	***26***		***594***	***50***	***26***		***221***	***89***	***8***		***221***	***56***	***20***	
Delivery timings				***0.000***				0.20				0.42				0.74				0.252
Step 1	**86**	**92**	**5**		126	50	22		126	53	21		54	89	7		54	54	17	
Step 2	**74**	**92**	**3**		146	44	26		146	48	26		44	88	9		48	53	23	
Step 3	**93**	**95**	**4**		158	48	27		158	50	28		56	90	6		60	56	23	
step 4	**98**	**93**	**5**		164	48	27		163	50	28		67	89	8		68	60	17	
Workplaces				***0.000***				***0.004***				***0.001***				***0.001***				0.125
Workplace 1	**112**	**95**	**3**		**172**	**53**	**28**		**172**	**57**	**27**		**73**	**92**	**5**		82	58	25	
Workplace 2	**115**	**91**	**5**		**194**	**44**	**25**		**194**	**47**	**26**		**73**	**88**	**8**		73	55	16	
Workplace 3	**30**	**94**	**2**		**56**	**48**	**22**		**56**	**51**	**23**		**20**	**86**	**11**		20	46	21	
Workplace 4	**94**	**94**	**4**		**171**	**46**	**25**		**171**	**47**	**26**		**55**	**89**	**7**		55	58	15	
Intervention components				***0.000***				***0.000***				***0.000***				***0.000***				***0.000***
Participatory ergonomics		**92**	**10**			**63**	**22**			**66**	**22**			**90**	**10**			**60**	**23**	
Physical training		**91**	**6**			**35**	**29**			**38**	**31**			**91**	**7**			**30**	**29**	
Cognitive behavioral training		**95**	**7**			**45**	**42**			**48**	**44**			**92**	**9**			**35**	**39**	

Units are percentages

SD standard deviation

Bold italic indicates significant *P* values at *P* <0.05

## Discussion

This study presents an operationalization of a quantifiable implementation evaluation suitable for interventions implemented at multiple organizational levels and with multiple components. It shows that implementation can be measured at both organizational level and at individual level, in a manner, that offers analyses across settings, timings, and components of interventions. The specific evaluation of the multifaceted intervention at hand shows that this effective intervention was delivered with a 91% success. Adding both fidelity and responsiveness to the participation measure reduces the impression of a highly implemented intervention and underlines the importance of more comprehensive implementation measures than just participation to understand the implementation. Finally, this study shows to which degree a stepped-wedge, multifaceted, multicenter intervention can be delivered with uniformity across timings but not workplaces and intervention components. First, the steps of the trial did not seem to introduce biasing learning effects in the intervention delivery. Second, the intervention components were delivered with varying success. Third, the delivery was dependent on workplaces. The different results of this study are discussed in relation to previous literature in the following.

### The operationalization of the quantifiable implementation evaluation

The operationalization presents a doable solution to quantifying implementation in a way that offers comparison with the intervention across components, timing of delivery, and settings. More in depth analyses can also be conducted, for example, to evaluate learning across sessions, differences in fidelity across deliverers, or to study the contribution of fidelity at the first sessions for participation in later sessions. However, most importantly, the quantifiable implementation measures can be used for analyzing the impact of implementation on the effectiveness of the study. For example, linear regressions can be performed between implementation and the primary outcome and perhaps even reveal if there are different impacts of different components of the intervention on the primary outcome. Previously, dose-response analyses have been performed in interventions, showing that higher dosage of an intervention was associated with higher effect [20]. However, the study did not allow for the comparison between intervention components and implementation was based on participation only. It is increasingly recognized that to obtain effectiveness in implementation studies, multiple effective components may be necessary and multiple implementation considerations are paramount. However, the more complex the intervention, the bigger the “black box” in the evaluation. Thus, by pre-defining and measuring each of the effective and implementation components, the content of the black box is revealed and the analyses of them may even offer knowledge that will change our understanding of effective and implementation components in multicomponent interventions.

### The evaluation of the implementation across delivery timing, intervention components, and workplaces

This study showed that using the stepped-wedge design was not a threat to the delivery and implementation of the intervention. Previous papers have discussed pros and cons for the stepped-wedge design and the risk of changed implementation of the intervention from step to step [14, 21]. Factors typically mentioned as threats to the uniform implementation are learning effects among the deliverers, organizational changes across time, or secular trends. However, these did not seem to impact the implementation of the current study.

The study showed some difference in the implementation of the three components; the CBT and PE were most highly implemented, whereas PT was least implemented as planned. This may indicate, that in a workplace setting, CBT and PE are easier to implement than PT. The reasons for this may be that participation is easier in CBT and PE sessions, which were delivered over a longer time slots of 3 h and thus required that the workplace logistic manager organized the logistics and called in substitute employees during the sessions leaving the participants with true “time off” from daily tasks. PT was conducted every week for 1 h and required ongoing continuous participation of the workers. Thus, for future implementation of such an intervention, consideration of how the PT should be delivered ought to be considered. However, to fully understand why PT was less well implemented, qualitative process evaluation methods could provide valuable knowledge.

There were some differences in the implementation across workplaces. Studying the different implementation measures reveals that both fidelity, exposure, participation, and responsiveness were different, but the overall implementation did not differ across workplaces. This finding again underlines the importance of separating components of implementation measures to gain insight into what hampers the implementation. The differences observed in implementation components across workplaces indicate that the contexts are of greater importance for the implementation than the actual intervention itself.

Limitations of this study include a tendency towards a general overestimation from the deliverers compared to the observer as found in the inter-rater reliability of the logbooks. The questions that we used may be influenced by intentional false reporting, inattentive responding, or memory limitations that introduce a bias. Therefore, data collection procedures should be considered when collecting data of fidelity. Furthermore, this study is explorative, and more trials of the quantifiable implementation evaluation will improve our understanding of the model for our understanding of implementation. For instance, the current model relies on a thorough preparation of a protocol by the main researchers, as given in the three steps in the methods section. That protocol is what for this model has determined the “optimal” implementation. However, whether the anticipation of the optimal implementation was correct and how the different implementation measures may interact needs further studies of these data. Moreover, operationalizing constructs is only one aspect of making a model testable. It also requires information about construct validity and knowledge about the generalizability of the model and which contexts or factors might limit its applicability. This warrants for further testing of the model.

## Conclusion

This study developed an operationalization of a quantifiable implementation evaluation measuring participation, fidelity, exposure, and responsiveness. The evaluation is suitable for interventions implemented at multiple organizational levels, where implementation can be measured at both organizational level and at individual level. The implementation evaluation was applied to a multifaceted intervention and showed that the intervention sessions were delivered with a 91% success (fidelity). The implementation of the interventions was uniform across steps, intervention components, and workplaces. However, participation, fidelity, exposure, and responsiveness varied between workplaces and intervention components. The quantifiable implementation evaluation can be applied in analyzing the impact of implementation on the effectiveness of complex interventions.
